# Ru^0^·Ru^**n**+^/Al_2_O_3_ as a Versatile Catalyst in the Isomerization of Allyl Alcohol

**DOI:** 10.3389/fchem.2021.671980

**Published:** 2021-05-04

**Authors:** Julián Enciso, Alfonso Ramírez, Carlos Ostos, Adriana Echavarría, Misael Córdoba, Cecilia Lederhos, Cristian Miranda

**Affiliations:** ^1^Grupo de Investigación en Catálisis, Departamento de Química, Universidad del Cauca, Popayán, Colombia; ^2^Grupo de Catalizadores y Adsorbentes, Departamento de Química, Universidad de Antioquia, Ciudad Universitaria, Medellín, Colombia; ^3^Instituto de Investigaciones en Catálisis y Petroquímica (INCAPE-Consejo Nacional de Investigaciones Científicas y Técnicas (CONICET)), Santa Fe, Argentina

**Keywords:** isomerization, allyl, atomic economy, green chemistry, ruthenium—catalyst

## Abstract

This study focuses on examining the isomerization of allyl alcohol using ruthenium (Ru) supported on alumina as a heterogeneous catalyst. The synthesized Ru/Al solids were characterized by various characterization techniques. The content of Ru was estimated by the energy dispersive x-ray technique. The x-ray diffraction (XRD) confirmed the presence of phases in the support and active species in the catalysts. The surface area of the support after Ru impregnation and the pore volume were determined by nitrogen physisorption. The analysis of programmed temperature (TPR and TPO) shows different redox sites which is confirmed by XPS. The catalytic results suggest a dependence on the amount of available metallic Ru, as well as the importance of the continuous regeneration of the metal using H_2_ to achieve a good conversion of the allyl alcohol. For comparison purposes, the commercial Ru on alumina 5% (CAS 908142) was used. The results show up to 68% alcohol conversion and 27% yield of the isomerization product using Ru_(1,5.4h)_/Al catalyst in comparison with 86% conversion and 39% yield of the isomerization product using CAS 908142. In contrast, our catalysts always presented higher TOF values (149–160) in comparison with CAS 908142 (101).

## Introduction

**Scheme 1:**
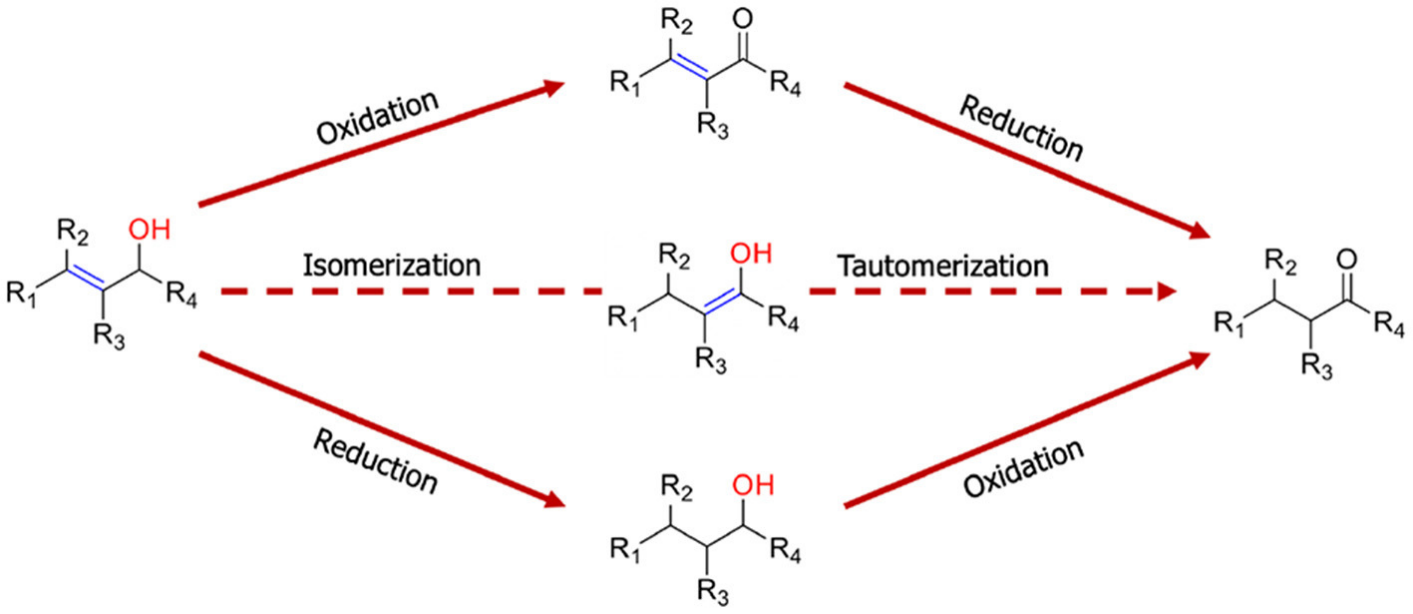
Saturated carbonyl compounds formation by redox and isomerization reactions.

Achieving a high atomic economy is essential in the search for new synthetic routes, adjusting the viability from the energy point of view, and being respectful of the environment. Obtaining saturated carbonyl compounds from allylic alcohols by isomerization is an example of this concept because conventional oxidation and reduction processes are avoided (Scheme 1). Saturated carbonyl compounds are of great industrial importance since they can act as organic synthetic intermediates (Shagufta and Panda, 2006).

To achieve the isomerization of allyl alcohol, the formation of the *enol* is essential first and then a tautomerization process is carried out. This process is easily accomplished with transition metal complexes, and therefore, several reports are found in the literature. These catalysts have proven to be very efficient and have a very varied selectivity depending largely on the catalyst used (Garcia-Alvarez et al., 2012). A general overview of transition metal complexes used was recently reported (Scalambra et al., 2019) and these include Ru (Lorenzo-Luis et al., 2012), Pd (Voronova et al., 2013), Rh (Czégéni et al., 2020), and Ir (Titova et al., 2017). Regarding the reports in the literature on the use of heterogeneous catalysts, they are less numerous, for example, gold clusters on La–Ni mixed oxides (Ishida et al., 2014), supported cationic rhodium (I) complex (Sahoo et al., 2012), palladium nanoparticles (Maung et al., 2017) amorphous copper-zirconium alloy (Martinek et al., 1996), PdAg nanoparticles (Moreno et al., 2012), and Ru(OH)_x_/Al_2_O_3_ (Kim et al., 2008). Some reports show that the selectivity of reaction can be influenced by different factors, namely, solvent (Yamaguchi et al., 2005; Hitrik and Sasson, 2018). One reason that fewer heterogeneous catalysts are used in this reaction than homogeneous catalysts may be due to the fact that it is critical to design a solid that is capable of adsorbing alcohol to the surface, and secondly, that this solid can promote isomerization. In this sense, Zsolnai et al. (2016) used Pd supported on alumina so that the support activated the allyl alcohol and, on the other hand, the palladium promoted a dehydrogenation oxidative with subsequent hydride transfer without the addition of H_2_ gas.

A possible alternative route to that described by Zsolnai et al. (2016), in addition to the proper use of alumina as a support due to its Lewis acid properties, could be to start from a metal that promotes partial hydrogenation of the double bond and subsequent rearrangement to form the *enol*. This would be achieved starting from a metal that is in its reduced form and contains hydride on its surface. In this sense, the present study proposes the use of Ru supported in alumina, under the presence of H_2_, to verify the formation of *n*-propanal from allylic alcohol. To examine whether the reduced form of the metal is capable of promoting the reaction, the reaction system is also compared under inert (N_2_) and oxidizing (air and O_2_) atmospheres.

## Experiment

### Catalyst Preparation

To obtain the solids, RuCl_3_.xH_2_O (Merck, US) was used as a ruthenium (Ru) precursor and γ-Al_2_O_3_ (CK 300 Ketjken) as a support, by means of the wet impregnation technique. Three different amounts of RuCl_3_.xH_2_O were taken with respect to the support (100 mg), in order to obtain percentages of 1.5, 1, and 0.7% of supported metal. Subsequently, the solids obtained were calcined at 400°C for 4 h in airflow and were reduced to 300°C in hydrogen flow varying the time (1 or 4 h). Additionally, commercial catalyst Ru on alumina 5% Ru (CAS 908142) was used.

### Catalyst Characterization

The Ru content was determined by wavelength dispersive x-ray fluorescence (WDXRF) using an ARL OPTIM'X spectrometer. The surface areas of catalysts were analyzed by low temperature N_2_ adsorption/desorption using a Micromeritics ASAP 2020 PLUS gas absorption analyzer and the specific surface areas were calculated using the Brunauer–Emmett–Teller (BET) equation and the pore volume using the *t*-plot method. The temperature-programmed reduction (H_2_-TPR), temperature-programmed oxidation (O_2_-TPO) were carried out on a chemisorption analyzer (Chemisorb 2720, Micromeritics, US) with 10 vol.% H_2_/Ar for TPR and 5% O_2_/He for TPO at a heating rate of 30°C/min from 50 to 600°C, for TPR and 50 to 800°C for TPO. Prior to reduction, the sample (100 mg) was treated in N_2_ at 150°C for 1 h. The x-ray diffraction patterns of samples were recorded on a Panalytical Empyrean diffractometer using CuKα (λ = 1.5404 Å; 40 kV y 40 mA). The patterns were recorded in the reflection mode at an angle 2θ between a range of 25° and 80° at a step size of 0.0260° 2θ and counting time of 50,215 s. The electronic state of surface species and their superficial atomic relationships were obtained by x-ray photoelectron spectroscopy (NAP-XPS). The measurements were made using a PHOIBOS 150 1D-DLD analyzer and a monochromatic source of Al-Kα (1486.7 eV, 13 kV, 100 W) with 90 eV step. The Ru 3d_5/2_ and 2p peak position of Cl and Al were followed. The areas of the peaks were estimated by calculating the integration of each peak after subtracting a Shirley background and fitting the experimental peak to a combination of Lorentzian/Gaussian lines of 30–70% proportions. The binding energy used as reference was the Al 2p at 74.7 eV, for the Al_2_O_3_ (NIST X-ray Photoelectron Spectroscopy Database, 2007).

### Isomerization of Allyl Alcohol

The catalytic tests were carried out in a glass autoclave under different atmospheres: H_2_ (reductive environment), N_2_ (inert environment), and air and O_2_ (oxidative environments). The temperature of the tests was programmed at 90°C and the pressure was adjusted to 1 bar for 10 h of reaction. The solvent used was toluene (10 ml) and the amount of allyl alcohol was 3.44 mmol, with 5% of catalyst (with respect to the amount of allyl alcohol). All catalytic tests were carried out in duplicate. Monitoring the reaction was carried out using a Shimadzu 14-A gas chromatograph equipped with a BaseLine®N-2000 chromatography data system (CDS), an RTX-5 chromatographic column, and a flame ionization detector. *n*-dodecane was used as an internal standard. The yield of the reaction products was calculated using the respective standard reagents.

The stability of the catalysts was evaluated by reuse as follows: (i) Once the first reaction cycle was finished, the catalyst was centrifuged, washed three times with 100 ml of deionized water, and dried at 80°C for 12 h. (ii) After the end of the second reaction cycle, the catalyst was recovered, washed, and dried as in step (i). (iii) Finally, the solid was reduced for 4 h at 350°C.

## Results

### Characterization of Solids

The results obtained by WDXRF (Table 1) show that the prepared solids presented a Ru content very close to the desired values (1.5, 1, and 0.7%), which indicates that the wet impregnation method was effective.

**Table 1:** Ru/Al based catalysts: commercial and synthesized.

**Catalyst^**a**^**	**% Ru**	**Reduction treatment**
Commercial	5%	WR^b^
C–Ru/Al		
Ru_(1,5.0h)_ /Al	1.5	WR
Ru_(1,5.1h)_ /Al	1.5	350°C during 1 h
Ru_(1,5.4h)_ /Al	1.5	350°C during 4 h
Ru_(0,9.4h)_ /Al	0.9	350°C during 4 h
Ru_(0,7.4h)_ /Al	0.7	350°C during 4 h

a *Code: Ru _(%Metal. Time of reductiontreatment)_/Al:Al_2_O_3_*.

b *WR, without reduction treatment*.

The values of the textural properties, the BET surface area and the pore volume are summarized in Table 2. According to the results of the surface area, it is observed that the 1.5% Ru catalysts have a higher surface area than the commercial catalyst. Regarding the pore volume, an impregnation with Ru leads to a decrease in the value of the initial pore volume of the support (Al_2_O_3_). This suggests that the deposition of metallic particles in the pores generated a blockage of the nitrogen adsorption sites. However, in impregnated solids with lower Ru content (0.9 and 0.7%), the surface value is higher than that of the support, probably due to the location of the metallic particles that are mainly dispersed on the outer surface of the support (Okal et al., 2007).

**Table 2:** Textural properties of solids: Al_2_O_3_, Ru/Al commercial and synthesized.

**Solid**	**S_**BET**_ (m^**2**^/g)**	**V_**p**_ (cm^**3**^/g)**
Al_2_O_3_	237	0.55
Commercial	93	0.26
C–Ru/Al		
Ru_(1,5.0h)_/Al	230	0.44
Ru_(1,5.1h)_/Al	234	0.45
Ru_(1,5.4h)_/Al	231	0.45
Ru_(0,9.4h)_/Al	245	0.49
Ru_(0,7.4h)_/Al	247	0.46

**Figure 1:**
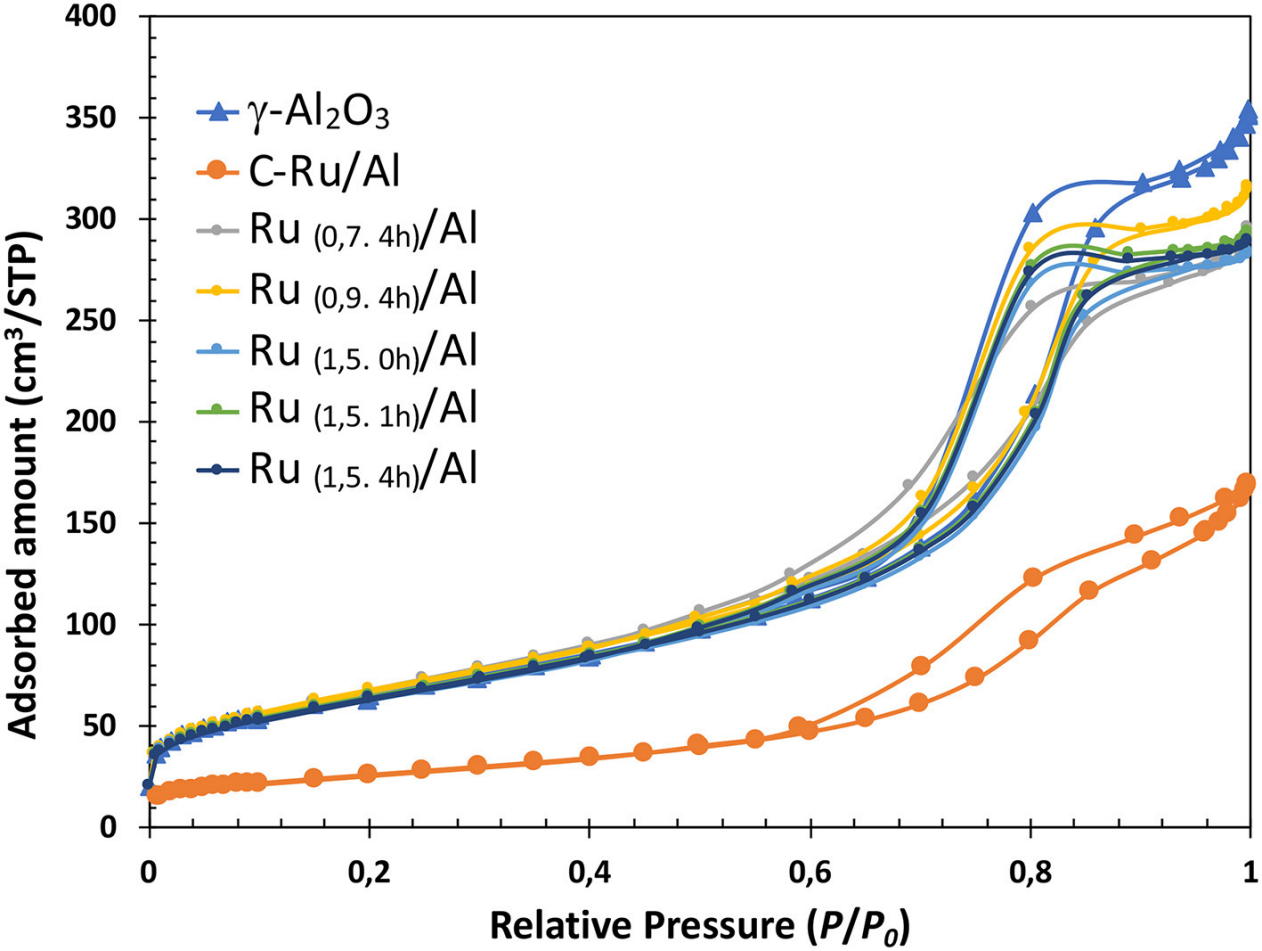
N_2_ adsorption isotherms (−196°C) of Al_2_O_3_, Ru/Al commercial and synthesized.

**Figure 2:**
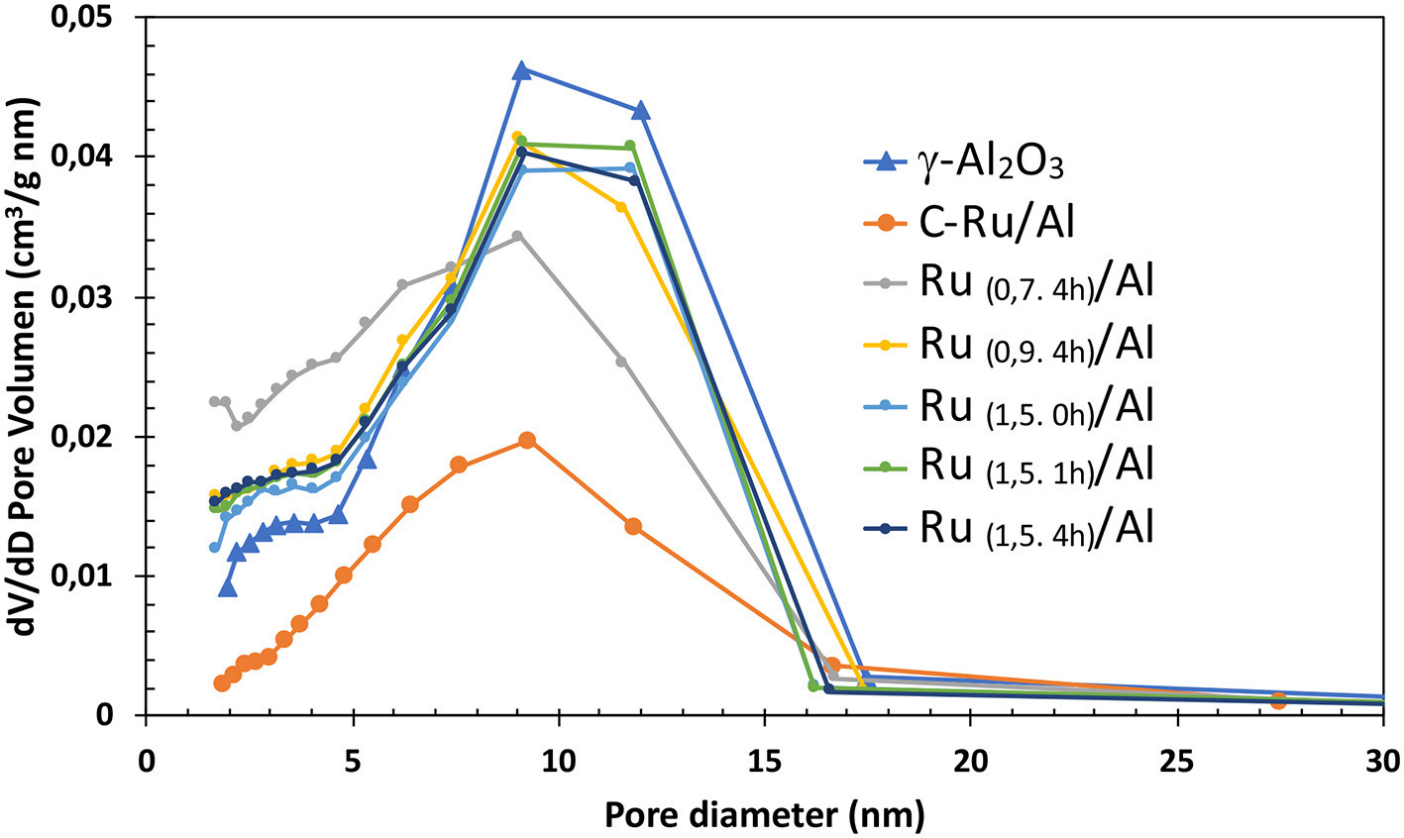
Solids pore size distribution: Al_2_O_3_, Ru/Al commercial and synthesized.

The nitrogen adsorption-desorption isotherms are shown in Figure 1, where a lower amount of nitrogen adsorbed by the commercial catalyst can be observed. In relation to the synthesized catalysts, it can be shown that the mesostructure is not influenced by the Ru load. The isotherms are maintained after impregnating the Ru, with respect to the isotherm of the support. In general, the profiles of the isotherms are related to a type IV mesoporous structure that retains a type H1 hysteresis loop. A homogeneous pore size distribution was found, 5–15 nm (Figure 2).

**Figure 3:**
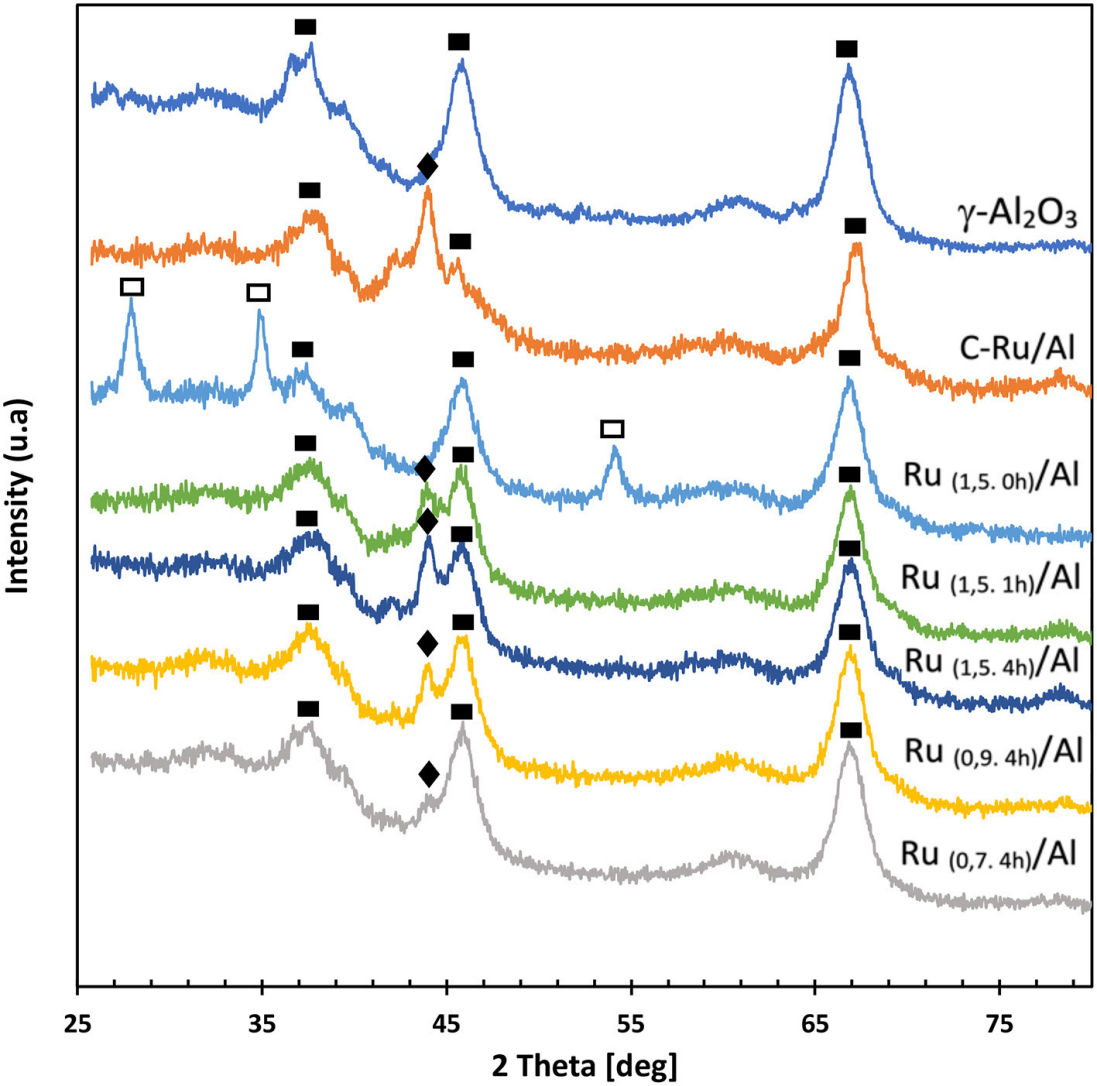
X-ray diffraction patterns supported Ru catalysts. (■) Al = γ-Al_2_O_3_; (□) RuO_2_; (♦) Ru.

The XRD patterns of the synthesized catalysts, the support, and the commercial catalyst are shown in Figure 3. According to the observed results, the characteristic peaks of γ-Al_2_O_3_ (ICDD 00-010-0425) are maintained in all solids after impregnation. Regarding the diffractogram of the calcined material [Ru_(1,5.0h)_/Al], diffraction peaks are shown that coincide with RuO_2_ (ICDD 00-040-1290) (Navarro-Jaén et al., 2019). In reduced solids, and even in the commercial catalyst, only the peaks corresponding to the reduced Ru are observed (Ru, 2Θ = 44°, ICDD 00.006-0663). For the reduced solids, it is also observed that the relative intensity of the Ru^0^ peak increases with the increase in the metallic charge and with the reduction time, indicating a greater presence of these species in the solid [Ru _(1,5.4h)_/Al].

**Figure 4:**
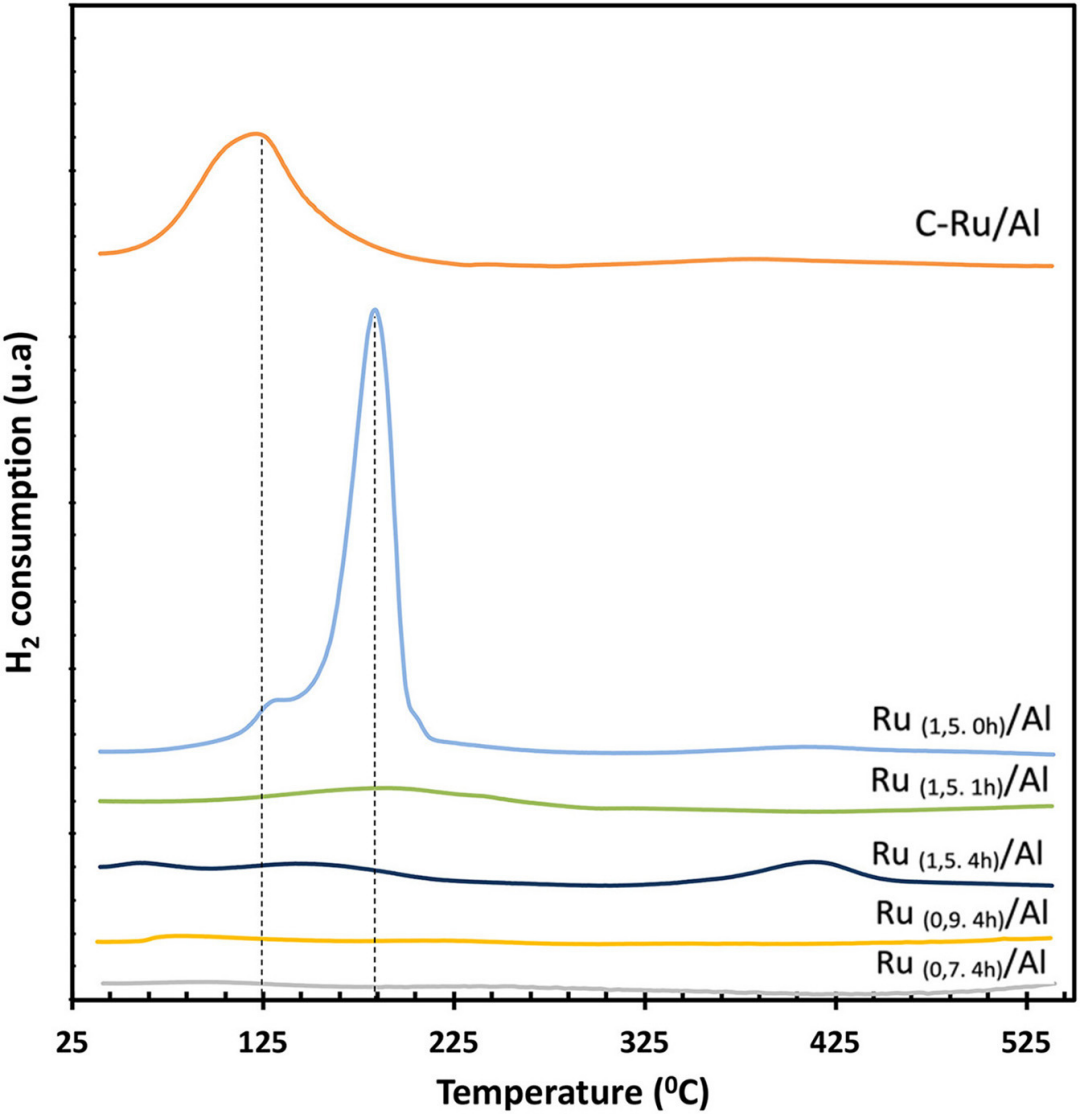
H_2_-TPR profiles for commercial and synthesized Ru/Al solids.

The results obtained from the analysis of programmed temperature reduction (H_2_-TPR) of the commercial catalyst (Figure 4) show a peak at 125°C that corresponds to the RuO_x_ species present on the surface. According to the results obtained by XRD (Figure 3), these species were not detected by diffraction, which indicates that RuO_x_ may be on the surface in the amorphous phase, highly dispersed, or as a thin film on top of the metallic Ru particles (Janina and Mirosław, 2009). On the other hand, the Ru_(1,5.0h)_/Al catalyst presents two peaks, at 125 and 170°C, which indicates two different oxidation states for Ru: RuO_x_ and RuO_2_, respectively (Mitsui et al., 2008; Li et al., 2009). The Ru_(1,5.1h)_/Al catalyst has a peak located at 184°C, which corresponds to the presence of RuO_2_, which indicates that for this solid there is Ru/RuO_2_ on the surface. For the Ru_(1,5.4h)_/Al, Ru_(0,9.4h)_/Al, and Ru_(0,7.4h)_/Al solids, there was no hydrogen consumption, indicating that there is no presence of oxidized species of Ru or that the amount present is minimal. Regarding the H_2_-TPR of the support, it is observed that it is not reducible in this temperature range.

**Figure 5:**
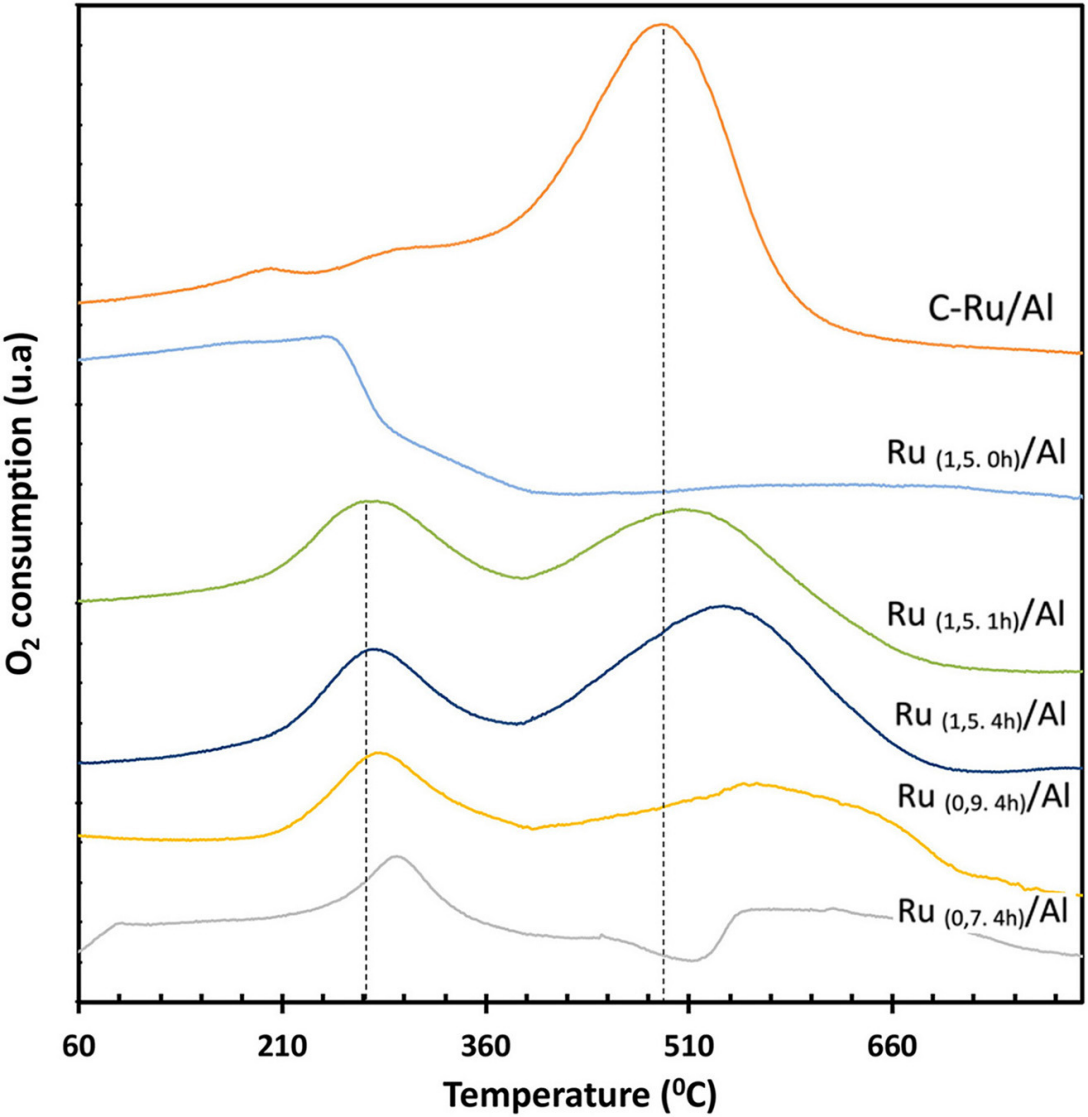
O_2_-TPO profiles for commercial and synthesized Ru/Al solids.

The TPO profiles for the catalysts are shown in Figure 5. For the solids Ru_(1,5.1h)_/Al, Ru_(1,5.4h)_/Al, Ru_(0,9.4h)_/Al, and Ru_(0,7.4h)_/Al, a peak is observed at ≈ 280°C, attributed to partially reduced Ru species. The TPO profile for all solids, except for the Ru_(1,5.0h)_/Al catalyst, shows a peak between 490 and 600°C, assigned to the metallic Ru (Balint et al., 2003). From the results obtained by TPR and TPO, it follows that the synthesized solids are composed of Ru^0^·Ru^n+^ supported on Al_2_O_3_.

**Figure 6:**
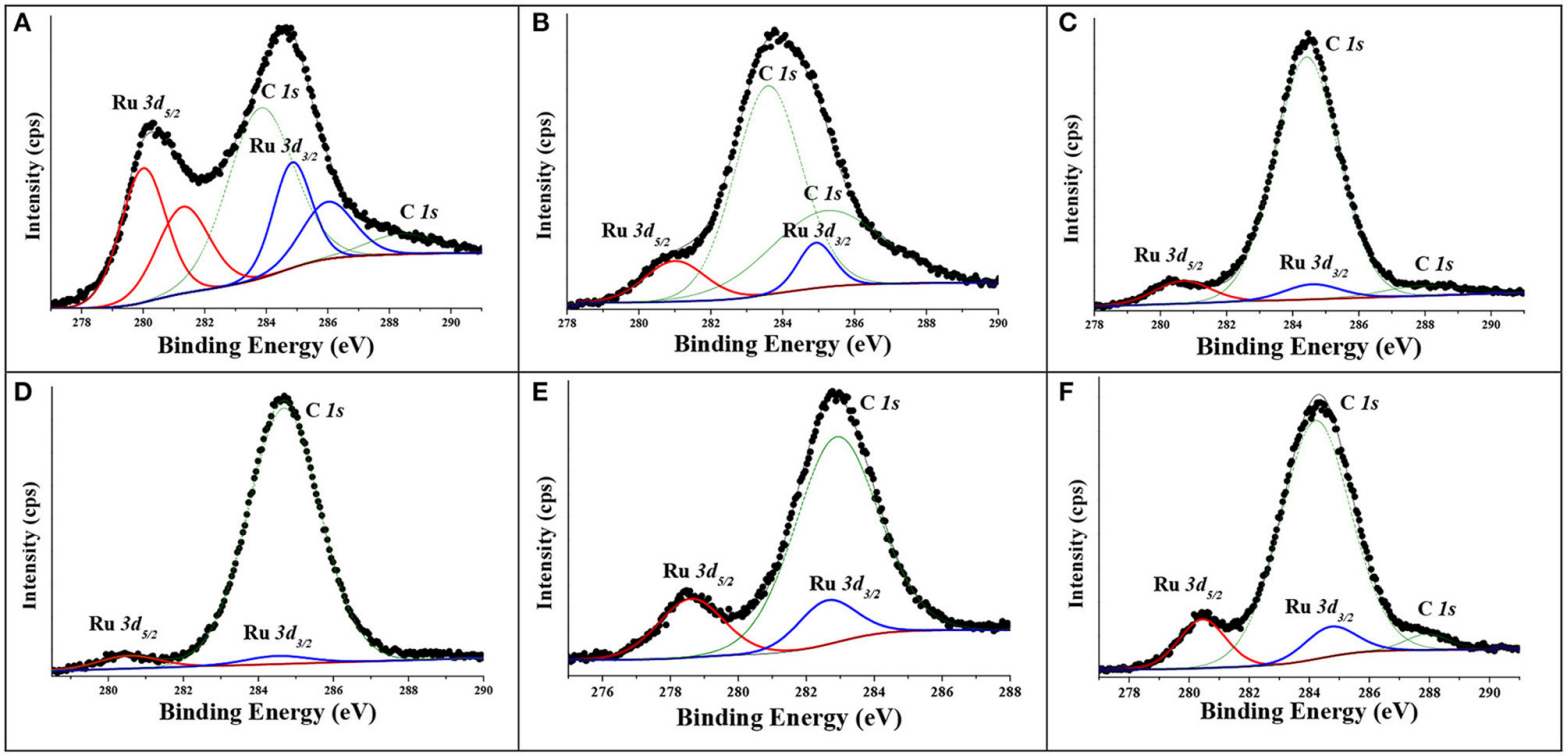
X-ray photoelectron spectroscopy spectra of ruthenium on alumina: **(A)** C–Ru/Al, **(B)** Ru_(1,5.0h)_/Al, **(C)** Ru_(1,5.1h)_/Al, **(D)** Ru_(1,5.4h)_/Al, **(E)** Ru_(0,9.4h)_/Al, and **(F)** Ru_(0,7.4h)_/Al.

After having resolved the Ru 3d doublet divided in spin orbit (Ru 3d_5/2_ and Ru 3d_3/2_) that appears in the same region of C1s, generating an overlap, the XPS spectra are shown in Figure 6. For the commercial catalyst, C–Ru/Al (Figure 6A) it is possible to observe Ru^0^ (280 eV) and oxidized species of Ru, indicated as RuO_x_ (281.3 eV) (Balcerzak et al., 2017). These results agree with those obtained in H_2_-TPR, where a broad peak was evident at 125°C. With respect to the Ru_(1,5.0h)_/Al catalyst, Figure 6B, only RuO_x_ (281 eV) is observed, corresponding to the heat treatment on this solid in air. On the other hand, the reduction treatment carried out on the supported Ru solids (Figures 6C–F) shows the presence of metallic Ru, being consistent with the results observed in XRD, H_2_-TPR, and even TPO.

### Catalytic Results

#### Conditioning Reaction

To establish the temperature and duration of the reaction at which a higher conversion of allyl alcohol was obtained, the commercial catalyst was used in a neutral atmosphere (N_2_). Initially, temperatures of 60, 75, and 90°C were evaluated for 40 continuous hours, observing that a temperature of 90°C and 10 h of reaction are sufficient to achieve a significant conversion. Additionally, the reaction carried out under the conditions of temperature, time, solvent, mechanical stirring, and without the use of a catalyst, did not generate conversion of the allyl alcohol.

**Scheme 2:**
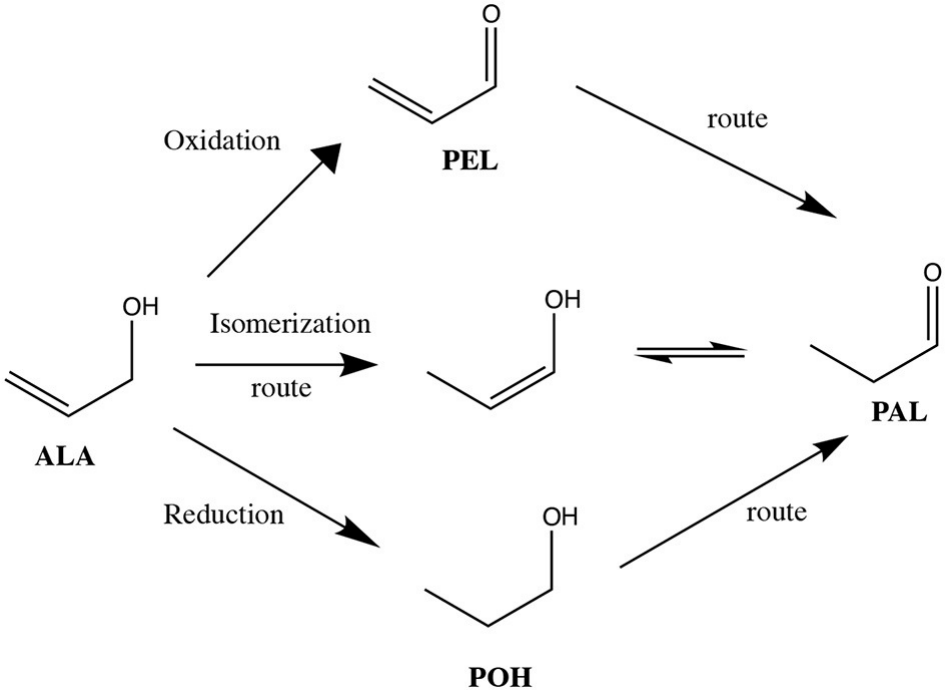
Transformation routes of allyl alcohol under redox and isomerization conditions. ALA, allyl alcohol; POH, *n*-propanol; PEL, propenal; PAL, *n*-propanal.

As the obtaining of *n*-propanal from allyl alcohol can be carried out by different synthesis routes (Scheme 1), the influence of the reaction atmosphere on the allyl alcohol, promoted by the same catalyst, was evaluated. Scheme 2 shows the different routes that can follow the allyl alcohol under experimental conditions.

**Figure 7:**
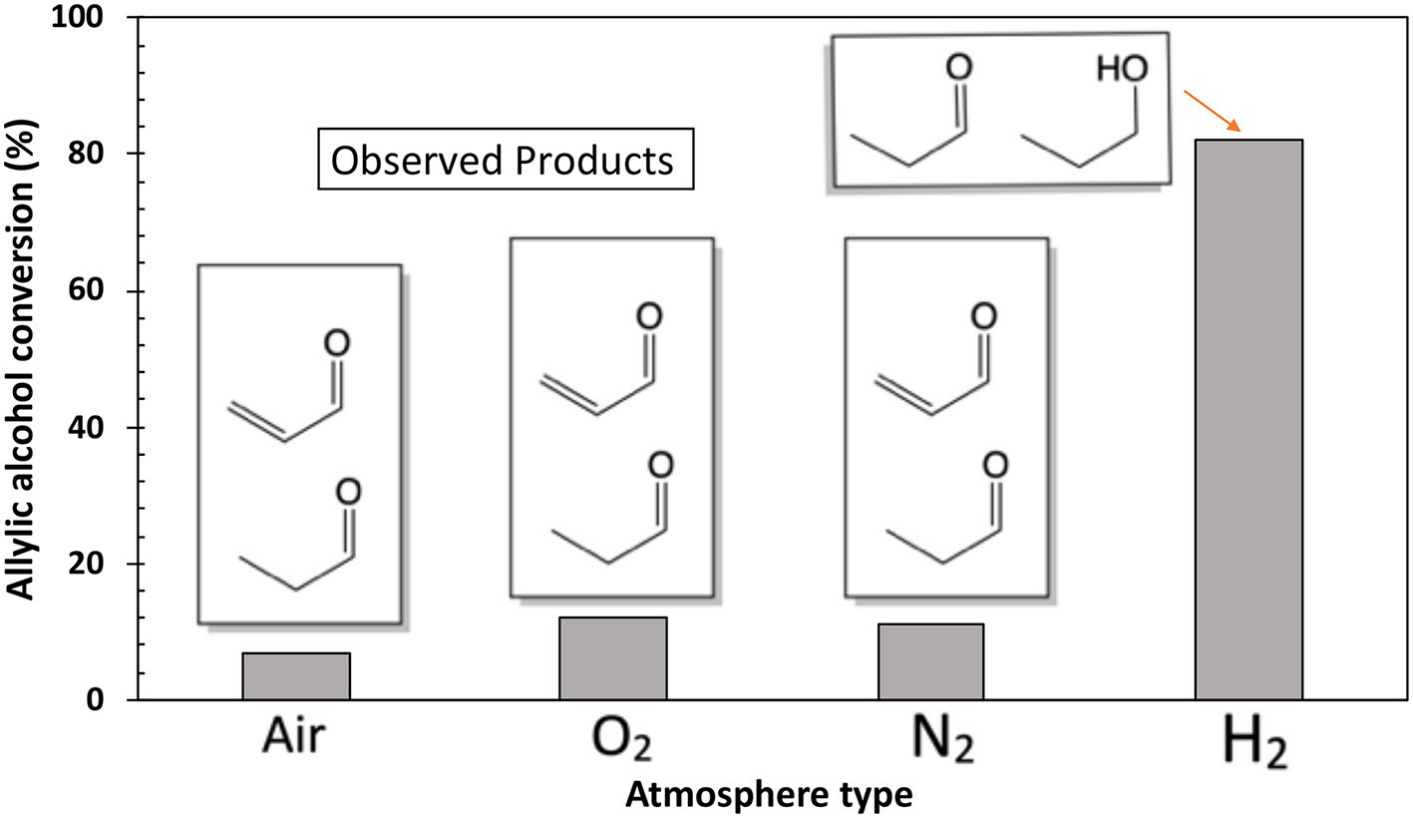
Atmosphere effect in allyl alcohol conversion. Reaction conditions: temperature of 90°C, 1 bar pressure, 10 h of reaction, 10 ml of toluene, 3.44 mmol of allyl alcohol, and 5% of the C–Ru/Al catalyst.

Figure 7 presents the effect of the atmosphere on the conversion of allyl alcohol in the presence of C–Ru/Al. According to the results, the PEL is only produced in an inert and oxidative environment, and POH only occurs in a reductive environment. Under reducing atmosphere (H_2_) a higher conversion is obtained (82%). These observations suggest that the behavior of the catalytic system depends on the atmosphere used in the reaction.

#### Catalytic Results

**Figure 8:**
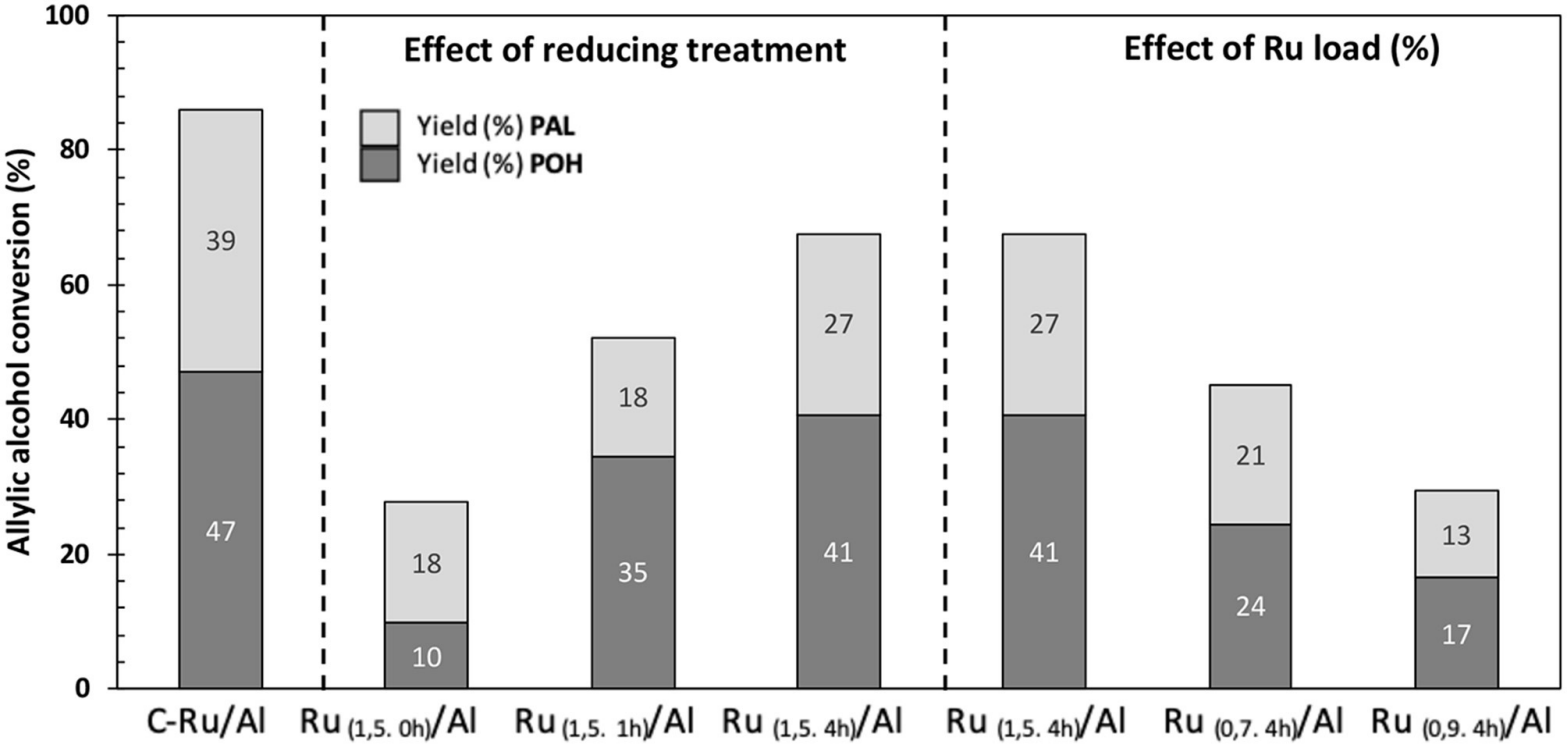
Effect of the reducing treatment and the ruthenium loading (%) of the Ru/Al catalysts on the conversion of allyl alcohol and the yield (%) of PAL and POH products.

Figure 8 shows the results of the effect of the reduction treatment and the metal loading of the solids on the conversion of allyl alcohol. Regarding the reduction treatment, Ru 1.5% supported in Al_2_O_3_ was compared, showing that this solid reduced for 4 h generates a higher conversion of alcohol (69%), followed by 1 h (55%), and finally, the catalyst not reduced [Ru_(1,5.0h)_/Al] shows a conversion of 28%. These results suggest that the high conversion of alcohol is related to a greater number of Ru–H species found on the surface. In this case, the main route followed is the direct hydrogenation of allyl alcohol to obtain propanol (reduction). In the case of the non-reduced catalyst [Ru_(1.5.0h)_/Al], which does not have reduced species of Ru, therefore the absence of hydride, the yield toward *n*-propanal was higher than that of propanol, suggesting that, in this case, the presence of hydroxyl groups (detected by IR, broadband between 3,000 and 3,700 cm^−1^) followed a mechanism that involved: (i) the formation of a ruthenium alcoholate by ligand exchange between the hydroxylated species of Ru and allyl alcohol and (ii) removal of ß-hydride to produce *n*-propanal and the corresponding species of ruthenium monohydride (Yamaguchi et al., 2005). In fact, the latter is corroborated with the use of the commercial catalyst under a neutral atmosphere and oxidants, in which the production of *n*-propanal was evidenced, despite not being in a reducing atmosphere (Figure 7). In the case of Ru_(1,5.4h)_/Al and Ru_(1,5.1h)_/Al solids, although the characterization did not show the presence of oxidized species of Ru (and therefore, cannot generate a mechanism similar to the case of Ru), they were able to generate *n*-propanal between the products. This is related to the ability of Ru by the addition and subsequent removal of hydride in allyl alcohol. In the next section, the heterogeneous mechanism for the isomerization of allyl alcohol by supported Ru is proposed.

**Figure 9:**
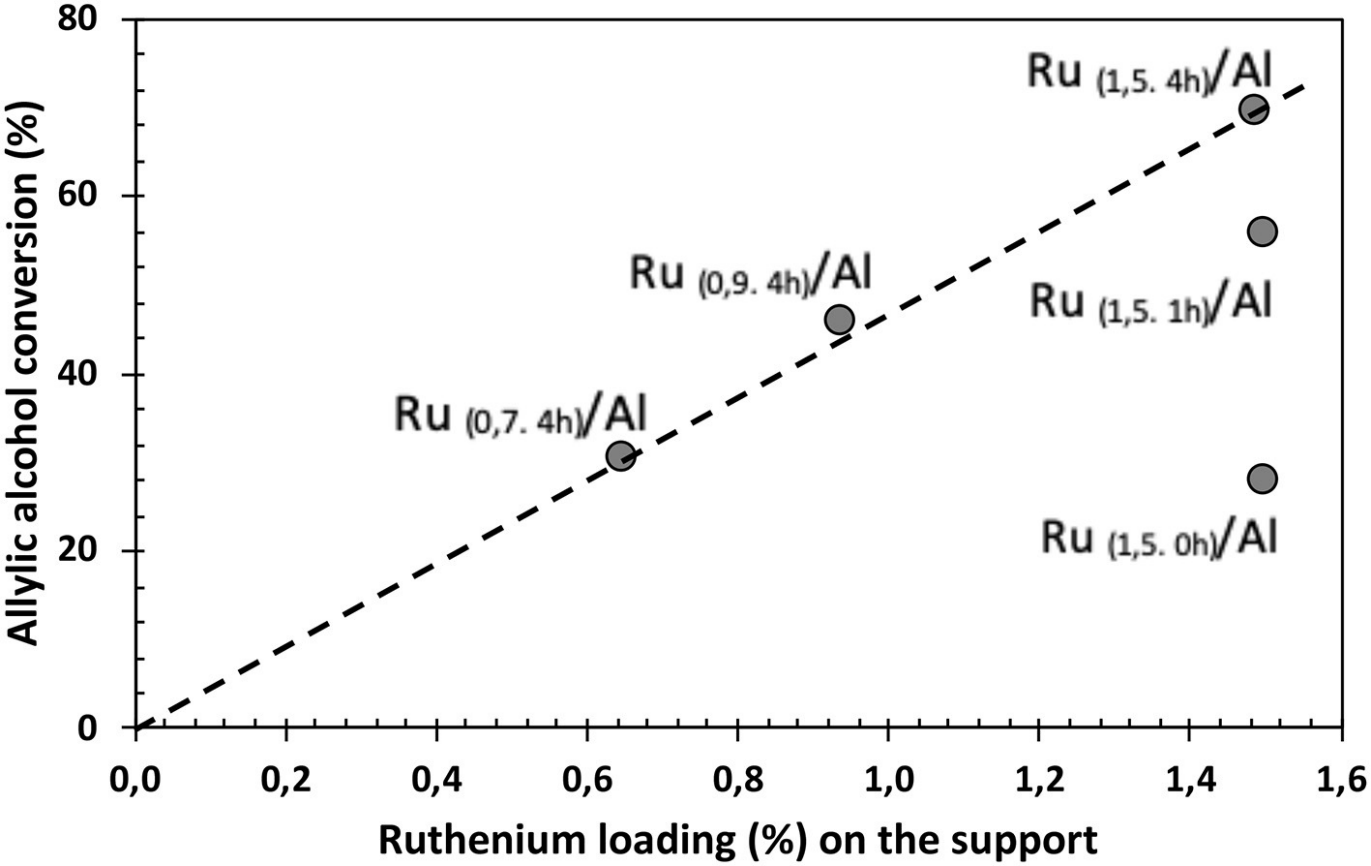
Correlation between the percentage of conversion of allyl alcohol and the loading (%) of Ru in the synthesized solids.

**Figure 10:**
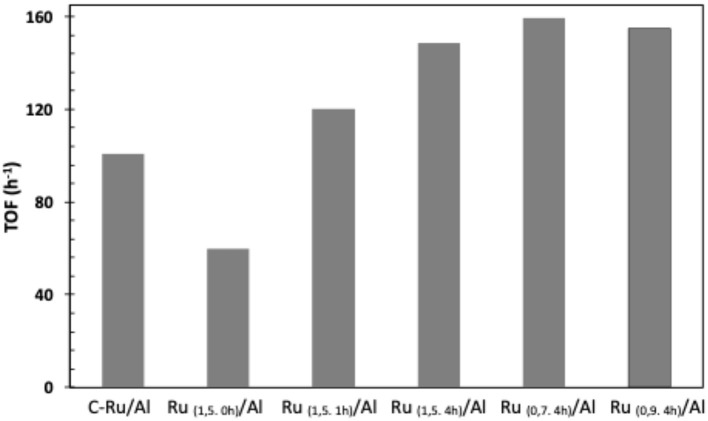
TOF for Ru/Al catalysts.

Regarding the effect of the percentage of Ru (Figure 8), a direct relationship is observed between the amount of Ru supported and the conversion of allyl alcohol, wherein by decreasing the amount of metal, the conversion also decreases, Ru_(1,5.4h)_/Al > Ru_(0,9.4h)_/Al > Ru_(0,7.4h)_/Al. This can be confirmed by the correlation between percent conversion and metal loading in solids (Figure 9). TOF values shows that our catalysts have a greater performance (Figure 10) than commercial Ru on alumina 5%.

**Figure 11:**
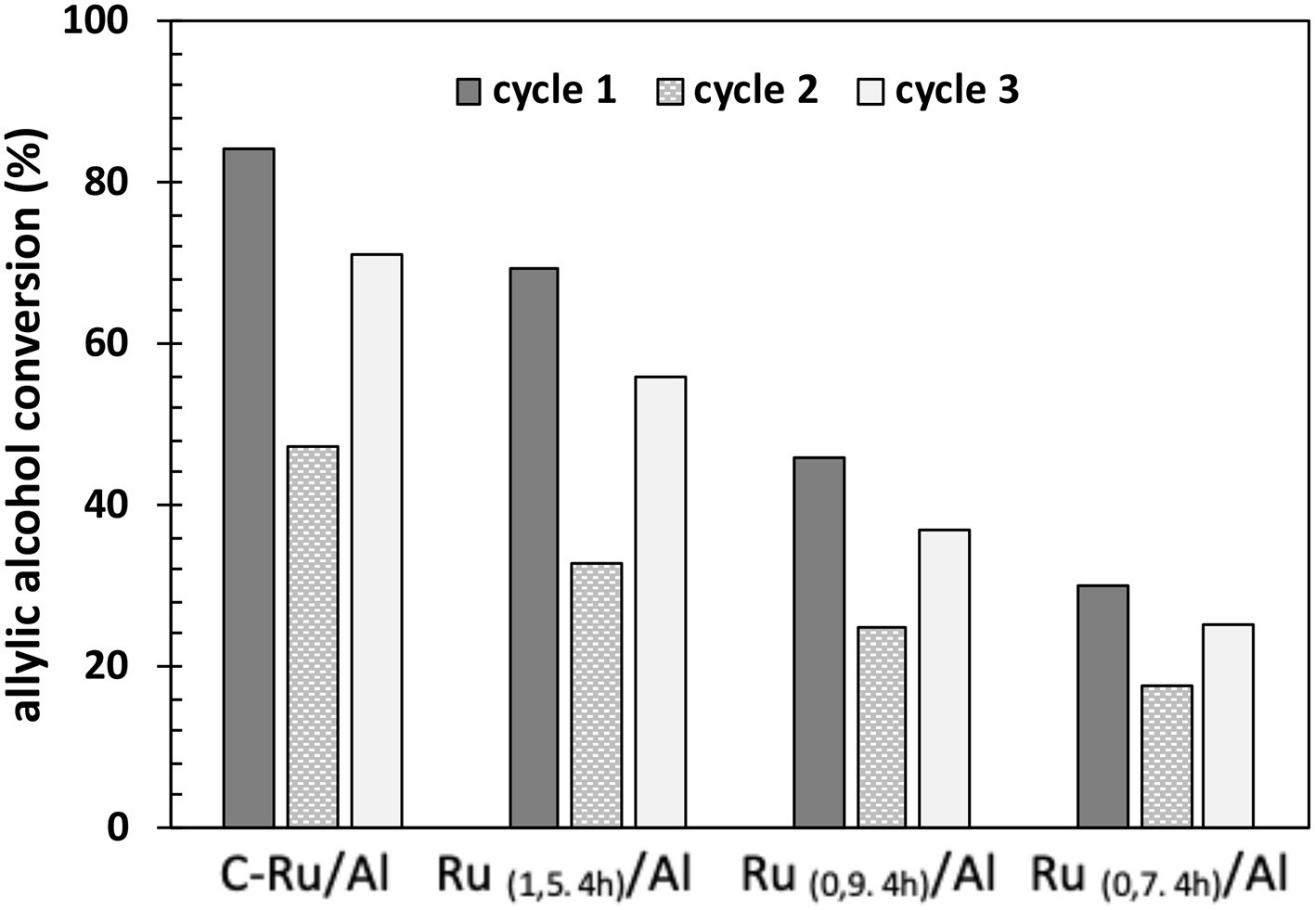
Evaluation of the stability of the catalysts based on Ru/Al, after reuse, followed by a reductive regeneration.

Additionally, the stability of the catalysts was evaluated (Figure 11). From the results obtained, it can be observed that the conversion of allyl alcohol decreases after the first cycle (cycle 1 to cycle 2), where only washes were carried out with water, and the solid was dried at low temperature (80°C). Therefore, after the completion of the reaction and the recovery of the solid, the Ru–H species were lost, and therefore, the conversion decreased. This is supported by the correlation shown in Figure 8. Additionally, after the second cycle, the catalyst was recovered and subsequent washing–drying, a reducing treatment was carried out. The conversion increased for all the catalysts, which shows that it is important to have Ru-H species to generate a good conversion of the allyl alcohol. The decrease (<9%) of the conversion by the catalysts of cycle three with respect to the first cycle, is mainly due to the desquamation of the metal on the Al_2_O_3_ support (Raut et al., 2016) or sintering effects (Morales-Cano et al., 2015).

#### Reaction Mechanism Proposal

**Figure 12:**
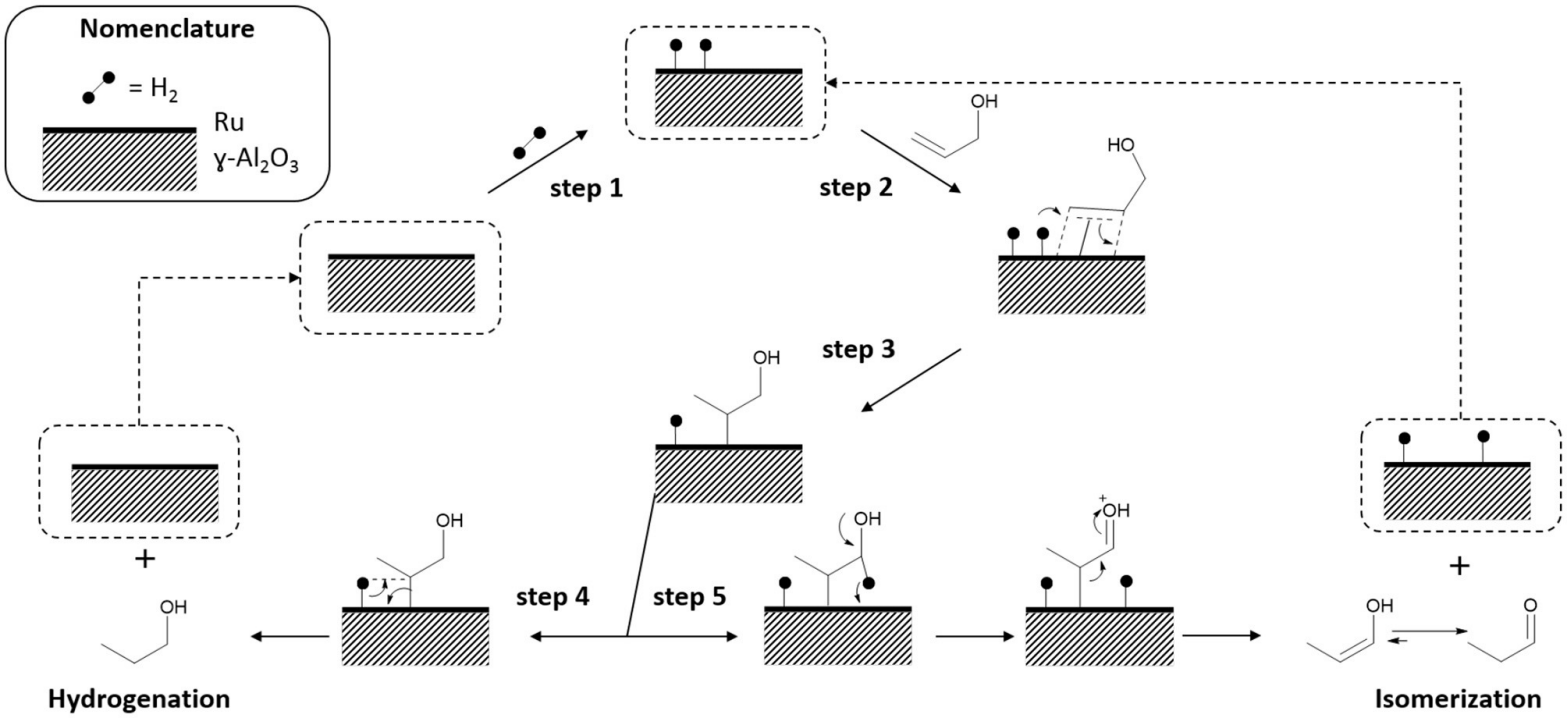
Proposed mechanism for the isomerization of allyl alcohol by Ru^0^Ru^n+^/Al_2_O_3_ catalyst.

From results, to obtain a high activity and formation of organic product by isomerization reaction the presence of hydrogen is important, indicating that mechanism involves, probably, the participation of hydride species formed from H_2_ adsorption on Ru. A common intermediary is also necessary to explain the formation of product by hydrogenation and isomerization reaction (Musolino et al., 2003). A reaction mechanism is proposed (Figure 12).

**Step 1:** A H_2_ molecule interacts with a metallic center, leading to a H–H homolysis (Hill, 2002).
**Step 2:** Allyl alcohol adsorption using π system from C=C bond according to Dewar–Chatt–Duncanson model (Iglesias, 2015).
**Step 3:** Alkyl–Ru intermediary formation (Horiuti and Polanyi, 1934).
**Step 4:** Formation of hydrogenation product (POH) from alkyl–Ru intermediary.
**Step 5:** Formation of hydrogenation product (PAL) from alkyl–Ru intermediary.

## Conclusions

Ru^n+^ supported on alumina can promote the isomerization of allyl alcohol, thanks firstly to a mechanism that involves the adsorption of allyl alcohol on the surface, secondly to the Lewis acid characteristics of the support, and thirdly to the formation of a ruthenium alcoholate by the exchange of ligands between the hydroxylated species of Ru and alcohol and the subsequent elimination of the ß-hydride. Ru^0^ supported on Al_2_O_3_, can also be formed by *n*-propanal, thanks to the Ru–H species on the surface. This last part was demonstrated in two ways: (i) prolonging the reduction time on the same percentage of supported Ru and (ii) increasing the metallic charge and reducing it under the same conditions. The continuous addition of hydrogen guarantees the conversion of allyl alcohol, by generating a homolytic decomposition of hydrogen, as demonstrated by the proposed mechanism. The oxidation state of Ru preferentially leads toward a specific pathway, wherein Ru^n+^ leads mainly toward isomerization, but Ru^0^ is able to hydrogenate alcohol mainly, although isomerization is also promoted. For this reason, Ru^n+^R^0^/Al_2_O_3_ can be considered as a versatile catalyst in the isomerization of allyl alcohol.

## Data Availability Statement

The raw data supporting the conclusions of this article will be made available by the authors, without undue reservation.

## Author Contributions

AR and CM conceived and designed the experiments, and analyzed the data. JE carried out the experiments. CO and AE characterized the samples using XPS. MC in collaboration with CL characterized the structure by TEM and XRD. This manuscript was written by AR and CM with input from CL and CO. All authors contributed to the article and approved the submitted version.

## Conflict of Interest

The authors declare that the research was conducted in the absence of any commercial or financial relationships that could be construed as a potential conflict of interest.
